# Factors Influencing the Clinical Adoption of Quantitative Gait Analysis Technologies for Adult Patient Populations With a Focus on Clinical Efficacy and Clinician Perspectives: Protocol for a Scoping Review

**DOI:** 10.2196/39767

**Published:** 2023-03-22

**Authors:** Yashoda Sharma, Lovisa Cheung, Kara K Patterson, Andrea Iaboni

**Affiliations:** 1 Rehabilitation Sciences Institute University of Toronto Toronto, ON Canada; 2 KITE - Toronto Rehabilitation Institute University Health Network Toronto, ON Canada; 3 Department of Physical Therapy University of Toronto Toronto, ON Canada; 4 Department of Psychiatry University of Toronto Toronto, ON Canada

**Keywords:** quantitative gait analysis, clinical adoption, clinical efficacy, clinician perspectives, barriers, facilitators, adults

## Abstract

**Background:**

Quantitative gait analysis can support clinical decision-making. These analyses can be performed using wearable sensors, nonwearable sensors, or a combination of both. However, to date, they have not been widely adopted in clinical practice. Technology adoption literature has highlighted the clinical efficacy of technology and the users’ perspective on the technology (eg, ease of use and usefulness) as some factors that influence their widespread adoption.

**Objective:**

To assist with the clinical adoption of quantitative gait technologies, this scoping review will synthesize the literature on their clinical efficacy and clinician perspectives on their use in the clinical care of adult patient populations.

**Methods:**

This scoping review protocol follows the Joanna Briggs Institute methodology for scoping reviews. The review will include both peer-reviewed and gray literature (ie, conference abstracts) regarding the clinical efficacy of quantitative gait technologies and clinician perspectives on their use in the clinical care of adult patient populations. A comprehensive search strategy was created in MEDLINE (Ovid), which was then translated to 4 other databases: CENTRAL (Ovid), Embase (Ovid), CINAHL (EBSCO), and SPORTDiscus (EBSCO). The title and abstract screening, full-text review, and data extraction of relevant articles will be performed independently by 2 reviewers, with a third reviewer involved to support the resolution of conflicts. Data will be analyzed using content analysis and summarized in tabular and diagram formats.

**Results:**

A search of relevant articles will be conducted in all 5 databases, and through hand-searching in Google Scholar and PEDro, including articles published up until December 2022. The research team plans to submit the final scoping review for publication in a peer-reviewed journal in 2023.

**Conclusions:**

The findings of this review will be presented at clinical science conferences and published in a peer-reviewed journal. This review will inform future studies designed to develop, evaluate, or implement quantitative gait analysis technologies in clinical practice.

**International Registered Report Identifier (IRRID):**

DERR1-10.2196/39767

## Introduction

Gait impairments increase risk of falls and the level of disability, thus negatively influencing a person’s involvement in society and quality of life. There are many causes of gait impairments, including neurological, musculoskeletal, and other health conditions. Gait assessments are an important aspect of a clinical assessment because they provide clinicians with information to support diagnosis, risk assessment, and treatment planning, with the goal of optimizing a person’s independence and physical function. In clinical practice, gait assessments are primarily performed using observational gait analysis, which involves a clinician’s visual assessment of the patient’s gait [1,2]. This analysis can also be supported by outcome measures such as the Functional Gait Assessment in the adult patient population [3]. However, the reliability of observational gait analysis has been questioned [4], with the amount of experience performing this analysis influencing reliability [5]. Additionally, observational gait analysis may lack precision in identifying the subtle nuances of gait that are important for treatment planning, understanding treatment outcomes, and clinical decision-making. Quantitative gait analysis can provide clinicians with objective measurements of the gait cycle that can help them assess and monitor changes in gait, thus better informing clinical decision-making. Quantitative gait analysis makes use of wearable sensors, nonwearable sensors, or a combination of both (eg, hybrid systems) [6]. Wearable sensors are attached to the patients themselves and allow for monitoring within or outside of a controlled environment [6-9]. Nonwearable sensors allow for the monitoring of gait within a controlled environment [6]. Examples of common wearable and nonwearable gait technologies are shown in Textbox 1.

**Common quantitative gait analysis technologies.**

**Wearable** [6-9]

- Inertial sensors (eg, accelerometers and gyroscopes)
- Force sensors (eg, instrumented insoles)
- Electromyography
- Flexible goniometers
- Ultrasonic sensors

**Nonwearable** [6,7]

- Pressure sensor mats
- Force plates (eg, ground reaction force plates)
- Motion capture
- Vision-based gait assessment [10]

Research on quantitative gait analysis technologies has increased nearly 10-fold over the last 10 years [11], with a nascent but expanding literature in support of the clinical efficacy of gait technologies. For example, 2 retrospective studies highlighted the role of gait technologies in guiding and optimizing treatment plans for people with stiff knee gait and spinal cord injury [12,13]. Both studies provide preliminary evidence for using gait technologies to enhance patient care. Moreover, guidelines for gait assessments [14-16] have been developed and can help support the use of these technologies in practice. However, despite this preliminary evidence of clinical efficacy, quantitative gait analysis technologies have yet to be widely adopted into clinical practice [2,17].

There are many factors that influence technology adoption [18-20], with evidence for clinical efficacy (ie, efficacy and effectiveness) only being a piece of the puzzle [20,21]. Instead, consideration must also be given to clinicians’ perspectives on using technology in their practice. The Technology Adoption Model suggests that 2 key factors influence users’ attitudes toward using a technology: perceived usefulness and perceived ease of use [18]. Perceived usefulness is defined as “the degree to which an individual believes that using a particular system would enhance his or her job performance” [18] (ie, perceived facilitator to usefulness) or the degree to which an individual believes that using the technology would not enhance his or her job performance (ie, perceived barrier to usefulness). Perceived ease of use is defined as “the degree to which an individual believes that using a particular system would be free of physical and mental effort” [18] (ie, perceived facilitator to ease of use) or the degree to which an individual believes that using the technology is not free of physical and mental effort (ie, perceived barrier to ease of use). Previous studies have used perceived usefulness and perceived ease of use to study technology acceptance in health care [22,23]. Beyond clinicians’ perspectives on ease of use and usefulness of technology, thought must also be given to their perspectives on factors that exist beyond ease of use and usefulness, such as whether they believe their workplace would support their use of the technology [20,24].

**Figure 1:**
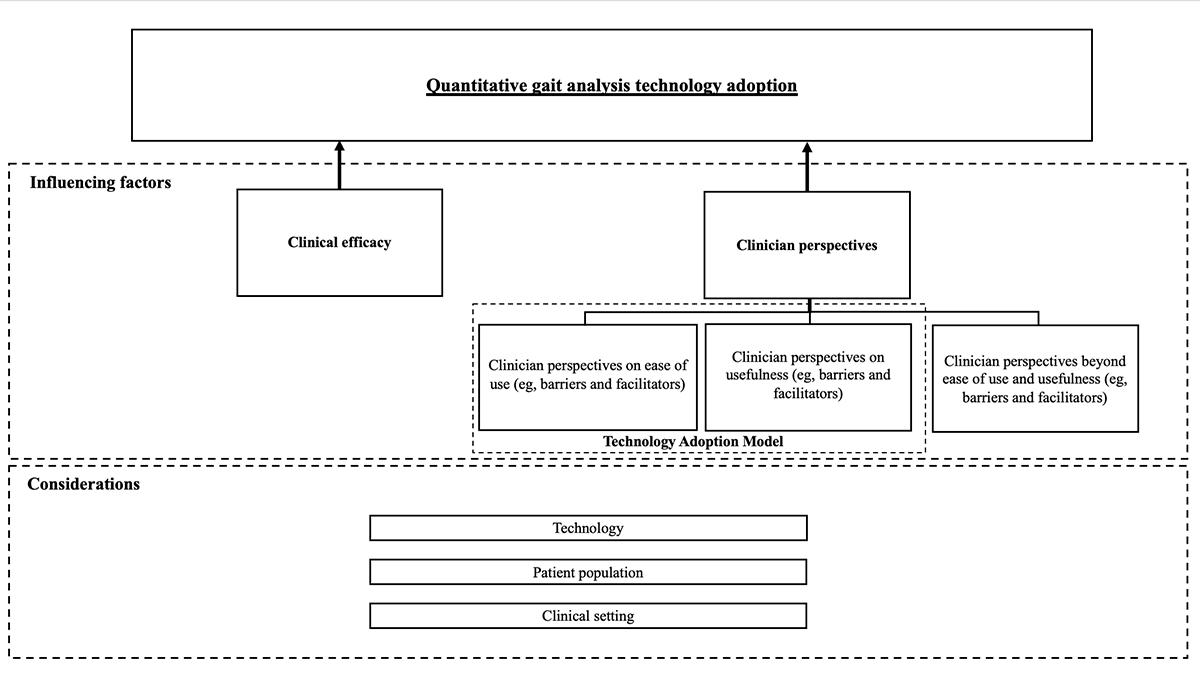
Factors influencing the clinical adoption of quantitative gait analysis technologies.

In applying these learnings to the clinical adoption of quantitative gait analysis technologies, we then consider some factors that influence their successful adoption to be clinical efficacy and effectiveness, and clinicians’ perspectives on the use of gait technologies in practice (ie, perceived barriers or facilitators to ease the use and usefulness of the technology, and beyond). As there are many different gait analysis technologies, consideration must be given to how and whether the above factors differ between each respective technology, patient population, and clinical context. A visual representation of how we consider these factors to influence the clinical adoption of quantitative gait technologies in the care of adult populations is shown in Figure 1.

To our knowledge, no previous review has synthesized the evidence for clinical efficacy and a clinician’s perspective that influences the use of gait technologies in the clinical care of adult patient populations. A preliminary search for similar existing scoping and systematic reviews was conducted up until April 2021 in PubMed and Google Scholar. Two related systematic reviews were published in 2011 [25,26]. Bergmann and McGregor [25] focused on clinician and patient preferences for wearable technologies. This review addressed wearable technologies broadly and did not examine gait analysis specifically. Wren et al [26] investigated the clinical efficacy of quantitative gait analysis in a review of studies up until 2009, which was recently updated in 2020 [11]. This review investigated the clinical efficacy of gait technologies across 6 categories: technical efficacy, diagnostic accuracy efficacy, diagnostic thinking, treatment efficacy, patient outcomes efficacy, and societal efficacy. However, this review did not consider clinicians’ perspectives on the ease of use and usefulness of gait technologies, and the recent update solely focused on 3D motion capture technologies.

The objective of this scoping review is to describe the factors influencing the clinical adoption of quantitative gait analysis technologies with a focus on their clinical efficacy, and clinicians’ perspectives on their use in the clinical care of adult patient populations. We will also explore how these components differ across gait technology types, patient populations, and clinical contexts. A scoping review approach has been chosen to explore the extent of literature on this broad topic and is a necessary step in identifying how gait technologies can be integrated into clinical practice.

## Methods

### Overview

This scoping review protocol follows the recommendations outlined in the Joanna Briggs Institute (JBI) methodology for scoping reviews [27].

The conduct and reporting of results will conform to the PRISMA-ScR (Preferred Reporting Items for Systematic Reviews and Meta-Analyses Extension for Scoping Reviews) checklist [28].

### Inclusion and Exclusion Criteria

A list of inclusion and exclusion criteria can be seen in Textbox 2.

**Inclusion and exclusion criteria.**

**Inclusion criteria**

- All adult patient populations (18 years and older)
- All health care professionals
- Quantitative gait analysis technologies
- Clinical efficacy studies (ie, efficacy and effectiveness)
- Clinician perspectives on the use of gait technologies in practice
- Clinical setting

**Exclusion criteria**

- Gait training technologies
- Gait technologies used solely to measure movement or activity levels
- Gait technologies in the development or preliminary validation phase

#### Types of Participants

There are 2 categories of participants included in this review. The first category of participants includes all adult patient populations (18 years and older), and the second category of participants includes all health care professionals. There will be no restrictions with regards to gender, sex, or ethnicity.

#### Concept

There are three components to the concept of this scoping review: (1) quantitative gait analysis technologies, (2) clinical efficacy (ie, efficacy and effectiveness), and (3) clinicians’ perspectives on the use of gait technologies in practice (eg, perceived barriers or facilitators to ease of use and usefulness of technology or factors beyond ease of use and usefulness). First, gait analysis technologies are defined as those used to quantitatively measure the different phases of gait for the purpose of assessment or monitoring changes in gait. This can include collecting kinetic or kinematic measures or muscle function data. By this definition, articles studying gait training technologies or technologies used solely to measure movement or activity levels (eg, number of steps per day) will be excluded. Additionally, studies focused on gait technology development or preliminary validation will be excluded from this review. Second, we define clinical efficacy similarly to the “diagnostic thinking and treatment efficacy” and “patient outcomes” categories as outlined by Wren et al [26]. This includes studies that investigate how clinicians use gait technologies (eg, treatment planning) with patient populations and what impact, if any, there is on patient outcomes. Both efficacy and effectiveness trials were included in this review. However, for simplicity, the term clinical efficacy will be used throughout this protocol. Third, clinicians’ perspectives on the use of gait technologies in practice encompass their perception of ease of use and usefulness of the technology and barriers or facilitators beyond ease of use and usefulness that they perceive to impact the use of gait technologies in practice. Barriers or facilitators beyond ease of use and usefulness include a clinician’s perception of how factors about themselves or their surroundings (eg, supportive environment, training, experience) influence the use of gait technologies in practice. The components of clinical efficacy and clinician perspectives do not need to occur within a given paper, but rather these components are combined using “OR” (eg, gait analysis technologies AND (clinical efficacy OR clinician perspectives)). How these components differ between the type of technology, patient population, and clinical context will also be examined in this review.

#### Context

There are no restrictions on geographical location or cultural factors in this review. The context of this scoping review includes “clinical care,” which encompasses a wide range of settings, including but not limited to hospitals, homes, community-based locations, and private health clinics. Because we are investigating clinician perspectives, it is likely that many of the articles will be qualitative in nature. Thus, the context of these papers may include interview or focus group settings.

#### Types of Evidence Sources

Primary research studies of all designs, including both quantitative and qualitative work, as well as gray literature (ie, conference abstracts), will be included in this review. Secondary research studies (eg, systematic reviews) and other gray literature (eg, textbooks and dissertations) will be excluded from this review. Additionally, articles and conference abstracts will be excluded if there are no reported results.

### Search Strategy

A 3-step search strategy was performed as outlined by the JBI methodology for scoping reviews [27]. First, a preliminary search of relevant sources consisted of hand-searching primary research articles that met the above inclusion criteria in PubMed and Google Scholar. Search terms included: Gait technologies AND clinician perspective, gait technologies AND clinical care, gait analysis AND barriers. A thorough analysis of the keywords included in the title and abstracts of selected articles was then conducted. Second, a comprehensive search strategy was created in MEDLINE (Ovid) based on the list of keywords and subject headings derived from the previous analysis and synonyms found by the first author. Boolean operators were used in the search strategy in two separate ways: (1) to combine all keywords that describe a single concept using “OR” and (2) to combine all 3 concepts using “AND” to develop a final search. The MEDLINE (Ovid) search strategy was created in collaboration with an information specialist at the University Health Network. Search terms were tested, and adjustments to the search strategy were made based on the identification of new keywords. Between March and April 2021, the final MEDLINE (Ovid) search strategy was translated to 4 other databases: CENTRAL (Ovid), EMBASE (Ovid), CINAHL (EBSCO), and SPORTDiscus (EBSCO). Engineering databases are excluded from this search because this review considers technology applications in a clinical context. The list of keywords and subject headings used in the MEDLINE (Ovid) search string was modified (eg, synonyms found) to suit the search capacity of the other databases. In the SPORTDiscus (EBSCO) database, subject headings from the MEDLINE (Ovid) search were omitted as no synonyms were found. The search will be iterative in nature, with adjustments being made to the search strategy in all databases as new keywords are identified. The third search strategy involved scanning the reference list of included literature and relevant review articles. To add to the breadth of the search strategy, supplemental searching through PEDro and Google Scholar was performed. If the full text of the included articles cannot be found, the reviewers will contact the primary authors for this information.

Only articles written in the English language will be included in this review because that is the language understood by the authors. Both peer-reviewed and gray literature (ie, conference abstracts) discussed in the above 3-step search strategy will be included in this review. Conference abstracts are included to increase the comprehensiveness of this review and will be searched in Embase (Ovid) and CENTRAL (Ovid). These 2 databases were chosen because conference proceedings are indexed in Embase (Ovid), with some also being found in CENTRAL (Ovid). There is no date limit on the articles included in this review, because to our knowledge, no other scoping review has addressed this specific question. An information specialist at the University Health Network was involved in the development and review of the search strategies created in all 5 databases. The complete MEDLINE (Ovid) search strategy can be found in Multimedia Appendix 1.

### Description of Source Selection

Covidence (Veritas Health Innovation) systematic review software will be used for the management of the articles identified in the search strategy. Prior to performing the formal selection process, pilot testing will be completed with 4 reviewers (YS, LC, AI, and KKP). In accordance with the JBI methodology for scoping reviews, 25 titles and abstracts will be chosen for the reviewers to screen based on the inclusion criteria previously mentioned [27]. Inconsistencies found during the pilot testing will result in further refinement of the inclusion criteria. The formal screening process will begin once there is a 75% or greater agreement among reviewers on the chosen 25 titles and abstracts [27].

During the formal screening process, 2 reviewers (YS and LC) will independently perform title and abstract screening. Both reviewers must agree on the inclusion of the article for it to be processed in the full-text review stage. Conflicts between the 2 reviewers at this stage will be resolved by a third reviewer (AI). Once title and abstract screening is complete, pilot testing for the full-text review stage will begin with the same 4 reviewers (YS, LC, AI, and KKP). Similar to the pilot testing for title and abstract screening, all 4 reviewers will independently read 5 full-text articles and assess them for inclusion. Any discrepancies during the pilot testing will result in further refinement of the inclusion criteria. The formal full-text review process will begin once 75% agreement or more is reached during pilot testing. Once the pilot testing is complete, both reviewers (YS and LC) will independently read the full-text articles for inclusion. Where articles are agreed upon for exclusion but the chosen exclusion criteria differ between reviewers, both reviewers (YS and LC) will decide on the most appropriate exclusion criteria by consensus. A third reviewer (AI) will resolve conflicts as they pertain to including and excluding articles. The complete review process will be illustrated using a flow diagram and included in the appendices of the scoping review. A short explanation regarding the inclusion and exclusion of sources of evidence will also be included in the appendices of the scoping review.

### Critical Appraisal

This review will also include a critical appraisal of all full-text articles included in this review. The critical appraisal will be used to assess the quality of the study design, methods, and analysis of the included literature. The JBI critical appraisal tools will be used, as this is recommended to authors who are conducting reviews following JBI guidelines [29]. JBI critical appraisal tools will be chosen based on the study design of the literature and may include checklists for cross-sectional studies, case control studies, case reports, case series, cohort studies, qualitative studies, quasi-experimental studies, and randomized controlled trials [30-33]. The JBI critical appraisal tools will be piloted by both reviewers (YS and LC) on 3 studies: a randomized controlled trial, a qualitative study, and a case report. The remaining articles will be divided between both reviewers; one will critically appraise the evidence source, and the other will review the appraisal for accuracy. The results of the critical appraisal will not be used to determine the inclusion or exclusion of literature in this review. This is because the authors consider including papers of all methodological quality to be valuable to the research community because it shows the state of the literature in this field and whether higher-quality evidence is warranted. No scoring system will be used during critical appraisal. Authors will report on the quality of the articles by sharing the results of the critical appraisal in tabular format in the final review. This will allow readers to see how each study attempted to limit bias in the design, conduct, and data analysis.

### Data Extraction

A draft data extraction form has been created in accordance with the JBI methodology for scoping reviews [27]. Information retrieved from the included sources will include the following as available: study characteristics (eg, authors, year of publication, location of where the article was published or conducted, aims or purposes, population or sample size, type of population or sample), methods or methodology (eg, tradition of inquiry or quantitative design), intervention (if any), and findings as they address the scoping review question (eg, clinical efficacy or clinician perspectives). In situations where conference abstracts are associated with a full text, the reviewers will only extract data from the full text. In cases where there is no full text associated with the conference abstract, the abstract itself will be used for data extraction.

The data extraction form will be piloted by both reviewers (YS and LC) on 3 articles to ensure that relevant information is being extracted. Pilot testing will involve both reviewers (YS and LC) extracting data from 3 articles. The first reviewer (YS) will check for consistency in the extracted information between both reviewers. Both reviewers will then meet to discuss whether additional modification of the data extraction form is needed. Recognizing that the development of the data extraction form is iterative, modifications may be made throughout the extraction process by both reviewers. For modifications to be made, both reviewers will need to agree upon the added information categories. If disagreements arise, a third reviewer (AI) will be involved. The included literature will be divided between both reviewers (YS and LC); one will complete data extraction and the other will review the extraction for accuracy.

### Analysis of the Evidence

The results of this scoping review will be synthesized and presented using both quantitative and qualitative methods. Quantitative methods will include counts of the number of unique clinical uses, clinician perspectives, types of gait analysis technologies used, patient populations, and clinical contexts. Qualitative methods will be used to organize the findings into Figure 1. We will use a combination of deductive and inductive content analysis in this review. Deductive content analysis will be used to code the literature, similar to the visual representation outlined in Figure 1. Inductive coding will be used to describe how and whether the clinical efficacy of gait technologies and clinician perspectives (eg, ease of use, usefulness, and factors beyond ease of use and usefulness) differ depending on the type of gait technology, patient population, and clinical context. This process involves coding the data, developing categories, and forming concepts [34]. The first author (YS) will be responsible for completing all stages of the content analysis process. NVivo (version 12, QSR International Pty Ltd) software will be used as the data management system for the qualitative content analysis. To ensure the trustworthiness of the analysis, the first author will provide justifications for how the literature was organized into the components outlined in Figure 1. The first author will also meet with the research team throughout all stages to receive team feedback for further refinement of the content analysis process.

### Ethical Considerations

This scoping review does not require research ethics board approval.

### Patient and Public Involvement Statement

There was no patient or public involvement in the development of this protocol.

## Results

Results of this proposed scoping review will be presented in both a tabular format and a diagram. The table will display the quantitative analysis of the results. Results of the critical appraisal for each study will be shared within the text of the scoping review as well as in tabular format. The diagram will display the qualitative results and be similar to what is shown in Figure 1. In summarizing the results both quantitatively and qualitatively, this review will highlight the gaps in the literature surrounding how gait analysis technologies are used in practice and what supports or hinders their clinical adoption.

A search of relevant articles will be conducted in all 5 databases and through hand searching in Google Scholar and PEDro, including articles published up until December 2022. The research team plans to submit the final scoping review for publication in a peer-reviewed journal in 2023. If we experience any delays to the timeline (eg, a greater number of papers to screen than expected), we will recruit others to assist with screening and data extraction.

## Discussion

### Impact

Preliminary research suggests gait analysis technologies have a role in clinical decision-making [12,13]. Despite this potential value, gait analysis technologies are not commonly found in clinical practice. This review will describe the factors that influence the clinical adoption of quantitative gait analysis technologies with a focus on clinical efficacy and clinicians’ perspectives on their use in the clinical care of adult patient populations. To be comprehensive, this review will also highlight how these components differ across gait technology types, patient populations, and clinical settings.

To our knowledge, this will be the first review to synthesize the evidence on the clinical efficacy and a clinician’s perspective on the use of gait technologies in the clinical care of adult patient populations. This review differs from related systematic reviews [11,25,26] for several reasons. First, this review focuses on gait analysis technologies (ie, wearable and nonwearable) and how these technologies impact clinical decision-making or patient outcomes. Lastly, this review also takes into consideration the perspectives of clinicians on the use of gait technologies in practice.

### Limitations

This review has some limitations. First, this review will only consider conference abstracts as a source of gray literature, and second, this review will only consider studies written in the English language. Thus, relevant articles may be missed if written in another language.

### Conclusions

We anticipate that this review will provide insights into the clinical adoption of quantitative gait analysis technologies by highlighting their role in clinical decision-making and clinician perspectives on using gait technologies in practice. The results of this review can also be used to inform and guide future work focusing on facilitating the translation of technologies into practice. Recognizing the role of this review in technology adoption and clinical sciences, the results will be disseminated at clinical science conferences and through publication in a peer-reviewed journal.

## Appendix

Multimedia Appendix 1 MEDLINE search string.

## Abbreviations

JBI: Joanna Briggs Institute
PRISMA-ScR: Preferred Reporting Items for Systematic Reviews and Meta-Analyses Extension for Scoping Reviews
