# Supplementary material for: Factors Influencing the Clinical Adoption of Quantitative Gait Analysis Technologies for Adult Patient Populations With a Focus on Clinical Efficacy and Clinician Perspectives: Protocol for a Scoping Review
Source: JMIR Res Protoc. 2023 Mar 22;12:e39767. doi: 10.2196/39767 (PMC10131694; doi:10.2196/39767)
Supplement: Multimedia Appendix 1 [file resprot_v12i1e39767_app1.docx]

**Multimedia Appendix 1**

**MEDLINE search strategy**

| 1. exp Gait/ |  |
| --- | --- |
| 2. exp Locomotion/ |  |
| 3. exp Walking/ |  |
| 4. gait.tw,kf. |  |
| 5. locomote.tw,kf. |  |
| 6. locomotion.tw,kf. |  |
| 7. ambulation.tw,kf. |  |
| 8. ambulate.tw,kf. |  |
| 9. walking.tw,kf. |  |
| 10. walk.tw,kf. |  |
| 11. stepping.tw,kf. |  |
| 12. 1 or 2 or 3 or 4 or 5 or 6 or 7 or 8 or 9 or 10 or 11 |  |
| 13. culturally appropriate technology/ or digital technology/ or educational technology/ or inventions/ or man-machine systems/ or medical laboratory science/ or quality control/ or technology transfer/ |  |
| 14. Accelerometry/ |  |
| 15. Fitness Trackers/ |  |
| 16. exp Foot Orthoses/ |  |
| 17. (technology or technologies).tw,kf. |  |
| 18. Technology/ |  |
| 19. (sensor or sensors).tw,kf. |  |
| 20. (body adj worn adj sensor?).tw,kf. |  |
| 21. wearable?.tw,kf. |  |
| 22. non-wearable?.tw,kf. |  |
| 23. (wearable adj device?).tw,kf. |  |
| 24. Wearable Electronic Devices/ |  |
| 25. (inertial adj sensor?).tw,kf. |  |
| 26. gyroscop*.tw,kf. |  |
| 27. (motion adj capture).tw,kf. |  |
| 28. ((floor adj sensor) or (floor adj sensors)).tw,kf. |  |
| 29. video?.tw,kf. |  |
| 30. camera?.tw,kf. |  |
| 31. (pressure adj sensor?).tw,kf. |  |
| 32. (pressure adj mat?).tw,kf. |  |
| 33. (gait adj mat?).tw,kf. |  |
| 34. (pressure adj platform?).tw,kf. |  |
| 35. accelerometer?.tw,kf. |  |
| 36. (instrumented adj insole?).tw,kf. |  |
| 37. (instrumented adj walkway?).tw,kf. |  |
| 38. electromyography.tw,kf. |  |
| 39. electromyogram.tw,kf. |  |
| 40. EMG.tw,kf. |  |
| 41. (wearable adj force adj1 plate?).tw,kf. |  |
| 42. (3D adj motion adj analysis).tw,kf. |  |
| 43. kinect.tw,kf. |  |
| 44. IMU?.tw,kf. |  |
| 45. (inertial adj measurement adj unit?).tw,kf. |  |
| 46. (motion adj analysis adj system?).tw,kf. |  |
| 47. (foot adj orthose?).tw,kf. |  |
| 48. 13 or 14 or 15 or 16 or 17 or 18 or 19 or 20 or 21 or 22 or 23 or 24 or 25 or 26 or 27 or 28 or 29 or 30 or 31 or 32 or 33 or 34 or 35 or 36 or 37 or 38 or 39 or 40 or 41 or 42 or 43 or 44 or 45 or 46 or 47 |  |
| 49. exp implementation science/ |  |
| 50. Pilot Project/ |  |
| 51. "Attitude of Health Personnel"/ |  |
| 52. exp Feasibility Studies/ |  |
| 53. implementation.tw,kf. |  |
| 54. (pilot adj project?).tw,kf. |  |
| 55. (pilot adj study).tw,kf. |  |
| 56. (pilot adj studies).tw,kf. |  |
| 57. implement?.tw,kf. |  |
| 58. implemented.tw,kf. |  |
| 59. (clinical adj uptake).tw,kf. |  |
| 60. barrier?.tw,kf. |  |
| 61. facilitate?.tw,kf. |  |
| 62. facilitator?.tw,kf. |  |
| 63. feasibility.tw,kf. |  |
| 64. acceptability.tw,kf. |  |
| 65. (clinic* adj3 acceptabl*).tw,kf. |  |
| 66. (clinical adj practice?).tw,kf. |  |
| 67. (clinical adj setting?).tw,kf. |  |
| 68. (clinical adj tool?).tw,kf. |  |
| 69. (clinical adj use*).tw,kf. |  |
| 70. (clinical adj context?).tw,kf. |  |
| 71. (clinical adj utility).tw,kf. |  |
| 72. (clinical adj adoption).tw,kf. |  |
| 73. (clinical adj application?).tw,kf. |  |
| 74. (Clinician? adj2 (perspective? or attitude? or preference? or view? or opinion? or belief?)).tw,kf. |  |
| 75. (physician? adj2 (perspective? or attitude? or preference? or view? or opinion? or belief?)).tw,kf. |  |
| 76. (physiotherapist? adj2 (perspective? or attitude? or preference? or view? or opinion? or belief?)).tw,kf. |  |
| 77. (healthcare adj provider? adj2 (perspective? or attitude? or preference? or view? or opinion? or belief?)).tw,kf. |  |
| 78. (healthcare adj personnel? adj2 (perspective? or attitude? or preference? or view? or opinion? or belief?)).tw,kf. |  |
| 79. (healthcare adj professional? adj2 (perspective? or attitude? or preference? or view? or opinion? or belief?)).tw,kf. |  |
| 80. (practitioner? adj2 (perspective? or attitude? or preference? or view? or opinion? or belief?)).tw,kf. |  |
| 81. (health adj personnel? adj2 (perspective? or attitude? or preference? or view? or opinion? or belief?)).tw,kf. |  |
| 82. (health adj practitioner? adj2 (perspective? or attitude? or preference? or view? or opinion? or belief?)).tw,kf. |  |
| 83. (health adj clinician? adj2 (perspective? or attitude? or preference? or view? or opinion? or belief?)).tw,kf. |  |
| 84. (health adj provider? adj2 (perspective? or attitude? or preference? or view? or opinion? or belief?)).tw,kf. |  |
| 85. (health adj care adj personnel? adj2 (perspective? or attitude? or preference? or view? or opinion? or belief?)).tw,kf. |  |
| 86. (health adj care adj practitioner? adj2 (perspective? or attitude? or preference? or view? or opinion? or belief?)).tw,kf. |  |
| 87. (health adj care adj clinician? adj2 (perspective? or attitude? or preference? or view? or opinion? or belief?)).tw,kf. |  |
| 88. (health adj care adj provider? adj2 (perspective? or attitude? or preference? or view? or opinion? or belief?)).tw,kf. |  |
| 89. (allied adj health adj professional? adj2 (perspective? or attitude? or preference? or view? or opinion? or belief?)).tw,kf. |  |
| 90. (allied adj health adj provider? adj2 (perspective? or attitude? or preference? or view? or opinion? or belief?)).tw,kf. |  |
| 91. (allied adj health adj practitioner? adj2 (perspective? or attitude? or preference? or view? or opinion? or belief?)).tw,kf. |  |
| 92. (allied adj health adj clinician? adj2 (perspective? or attitude? or preference? or view? or opinion? or belief?)).tw,kf. |  |
| 93. (allied adj health adj personnel? adj2 (perspective? or attitude? or preference? or view? or opinion? or belief?)).tw,kf.  94. exp Decision Making/  95. exp Clinical Decision Making/  96. (clinical adj decision-making).tw,kf.  97. (clinical adj decision adj making).tw,kf. |  |
| 98. 49 or 50 or 51 or 52 or 53 or 54 or 55 or 56 or 57 or 58 or 59 or 60 or 61 or 62 or 63 or 64 or 65 or 66 or 67 or 68 or 69 or 70 or 71 or 72 or 73 or 74 or 75 or 76 or 77 or 78 or 79 or 80 or 81 or 82 or 83 or 84 or 85 or 86 or 87 or 88 or 89 or 90 or 91 or 92 or 93 or 94 or 95 or 96 or 97 |  |
| 99. 12 and 48 and 98 |  |
| 100. limit 99 to english language |  |
| 101. 100 not ((exp child/ or exp infant/ or adolescent/) not exp adult/) |  |
| 102. 101 not (exp animals/ not humans.sh.) |  |
